# Anthropometrically estimated calf muscle circumference is a marker for early detection of muscle mass decline in older adults: Second report

**DOI:** 10.1016/j.jnha.2025.100729

**Published:** 2025-11-22

**Authors:** Ryo Sato, Yohei Sawaya, Tamaki Hirose, Takahiro Shiba, Lu Yin, Shuntaro Tsuji, Tomohiko Urano

**Affiliations:** a Integrated Facility for Medical and Long-Term Care, Care Facility for the Elderly “Maronie-en”, 533-4 Iguchi, Nasushiobara 329-2763, Tochigi, Japan; b Nishinasuno General Home Care Center, Department of Day Rehabilitation, Care Facility for the Elderly “Maronie-en,” Tochigi, Japan; c Department of Physical Therapy, School of Health Sciences, International University of Health and Welfare, Tochigi, Japan; d Department of Geriatric Medicine, School of Medicine, International University of Health and Welfare, Chiba, Japan

## 1 Introduction

Sarcopenia is a skeletal muscle disorder characterized by age-related declines in muscle mass and strength. Rapid progression of sarcopenia further increases the risk of frailty, disability, and cardiorespiratory multimorbidity [1,2]. Therefore, monitoring sarcopenia progression and implementing early interventions to slow its progression are critically important. However, skeletal muscle mass cannot be efficiently measured in facilities or home environments that lack specialized equipment. Therefore, we report that sarcopenia diagnosis using anthropometrically estimated calf muscle circumference (CMC) showed a high concordance rate with sarcopenia diagnosis using SMI [3]. However, it remains unclear whether changes in anthropometrically estimated CMC over time are associated with changes in skeletal muscle mass. Therefore, we aimed to clarify the relationship between changes in anthropometrically estimated CMC and changes in SMI.

## 2 Methods

This single-center, longitudinal study was conducted between September 2023 and March 2025. The follow-up duration was 18 months. The study protocol was approved by the Ethics Review Committee (Approval Numbers: 21-Io-22-3 and 17-Io-189-7), and all participants (or their families) signed informed consent forms. This study was conducted according to the principles of the Declaration of Helsinki. It involved 46 individuals (26 males, 20 females, mean age 80.7 ± 7.0 years) who required long-term care and attended day care centers for older adults. The exclusion criteria were as follows: (1) participants aged <65 years, (2) participants aged ≥100 years, (3) participants requiring walking assistance, (4) participants who refused to answer the SARC-F questions, (5) participants unable to be followed-up for 18 months, and (6) participants with missing data. Walking speed was measured twice, and the average was used as the representative value. Grip strength was measured twice in each hand using a dynamometer (TKK5401 Grip-D; Takei Kiki, Niigata, Japan), with the maximum value used as the representative value. SMI was calculated using a body composition analyzer (InBodyS10; InBody, Japan). Screening for sarcopenia was conducted using calf circumference, SARC-F, and SARC-CalF scores. Calf circumference was measured once on each side using a tape measure and recorded in millimeters, with the maximum value taken as the representative value. The measurement site was the area of maximum expansion in the seated position with the knee flexed. skinfold thickness was measured using a skinfold caliper (Abbott Japan Co., Ltd., Tokyo, Japan) by pinching the skin on the ventral side of the lower leg to the area of maximum expansion, thereby measuring the subcutaneous thickness. Based on these measurements, calculations were performed using the anthropometrically estimated CMC based on calf circumference measurement formula as a reference, with the maximum value serving as the representative value. The following formula was used to calculate CMC: Estimated CMC (cm) = Calf circumference (cm) – Subcutaneous fat thickness (mm) × π/10 (calculated using π = 3.14). The correlation between the changes in each variable was assessed using Spearman's or Pearson's correlation coefficient. A multiple regression analysis considering multicollinearity among factors related to SMI progression was conducted, with ΔSMI as the dependent variable and Δ estimated CMC as the independent variable.

## 3 Results

**Fig. 1:**
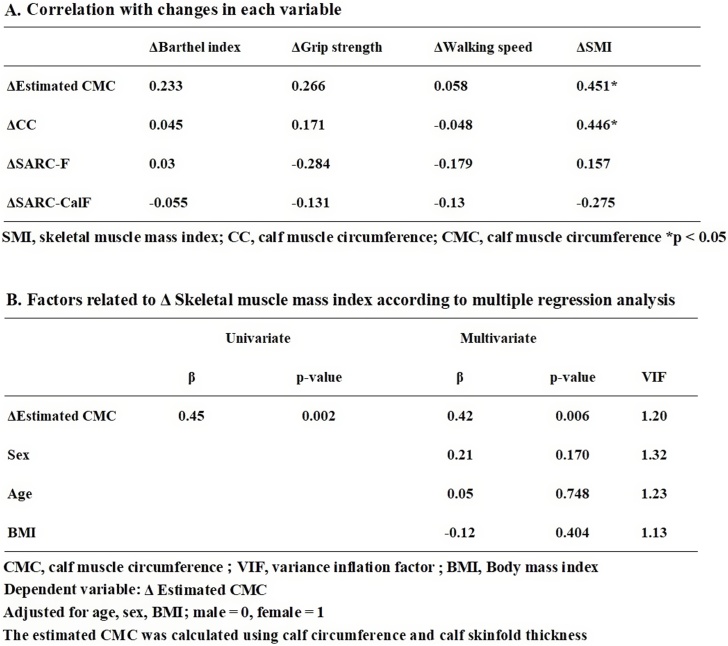
(A) Correlation with changes in each variable. (B) Factors related to Δ Skeletal muscle mass index according to multiple regression analysis.

Longitudinal analysis of factors associated with the changes in SMI revealed that ΔSMI showed a significant positive correlation with Δ estimated CMC (r = 0.451) and Δ calf circumference (r = 0.446) (Fig. 1A). Multiple regression analysis showed that Δ estimated CMC (β = 0.42, p = 0.006) was independently and significantly associated with ΔSMI. (Fig. 1B).

## 4 Discussion

This study evaluated the relationship between changes in estimated CMC and SMI using 18-month follow-up data. An independent association was observed between changes in SMI and estimated CMC. Previous cohort studies have reported that changes in calf circumference correlate with changes in SMI [4]. Herein, a positive correlation was also observed between calf circumference and the SMI course, supporting the findings of prior research. Interestingly, in the present study, changes in estimated CMC showed a positive correlation with changes in SMI compared with existing sarcopenia screening tools such as calf circumference, SARC-F, and SARC-CalF. Our previous results showed that estimated CMC correlated significantly with SMI but not with body fat percentage. In other words, estimated CMC is a screening method that is less susceptible to the influence of body fat [5]. Therefore, changes in estimated CMC may be associated with changes in skeletal muscle index a degree equivalent to or greater than existing screening tools. This is a preliminary study, and further multi-center collaborative research is needed.

## CRediT authorship contribution statement

**Ryo Sato:** Conceptualization, Data curation, Formal analysis, Investigation, Methodology, Project administration, Methodology, Project administration, Validation, Visualization, Writing – original draft. **Yohei Sawaya:** Conceptualization, Data curation, Funding acquisition, Investigation, Methodology, Project administration, Methodology, Project administration, Supervision, Validation, Visualization, Writing – original draft. **Tamaki Hirose:** Conceptualization, Data curation, Investigation, Validation, Visualization, Writing – original draft. **Takahiro Shiba:** Conceptualization, Data curation, Investigation, Validation, Visualization, Writing – original draft. **Lu Yin:** Conceptualization, Data curation, Investigation, Validation, Visualization, Writing – original draft. **Shuntaro Tsuji:** Conceptualization, Data curation, Investigation, Data curation, Investigation, Validation, Visualization, Writing – original draft. **Tomohiko Urano:** Conceptualization, Data curation, Funding acquisition, Investigation, Methodology, Project administration, Methodology, Project administration, Supervision, Validation, Visualization, Writing – original draft.

## Funding

This study was supported by the Japan Society for the Promotion of Science Grants-in-Aid for Scientific Research (grant numbers 23K06873, and 22K17539). The funding source had no involvement in the study design; in the collection, analysis and interpretation of data; in the writing of the report; and in the decision to submit the article for publication.

## Declaration of competing interest

The authors declare no conflict of interest.

## Glossary

SARC-CalF: SARC-F questionnaire combined with calf circumference measurement
SARC-F: a five-question questionnaire regarding strength, assistance walking, rising from a chair, climbing stairs, and falls
Sarcopenia: age-related loss of skeletal muscle mass, physical function, and muscle strength
